# Sudden Cardiac Arrest in Patient With Ventricular Septal Defect and Marijuana Consumption: A Case Report and Review of Literature

**DOI:** 10.7759/cureus.38113

**Published:** 2023-04-25

**Authors:** Mukosolu F Obi, Vikhyath Namireddy, Kelash Kumar, Zola N'Dandu, Cho Hyun

**Affiliations:** 1 Internal Medicine, Wyckoff Heights Medical Center, Brooklyn, USA; 2 Medicine, St. George's University School of Medicine, True Blue, GRD; 3 Internal Medicine, Maimonides Medical Center, Brooklyn, USA; 4 Interventional Cardiology, Ocshner Medical Center, New Orleans, USA

**Keywords:** sudden cardiac death (scd), ventricular tachycardia (vt), qt interval prolongation, premature ventricular contractions, ventricular septal defect (vsd)

## Abstract

This case report presents a detailed analysis of a 28-year-old woman who experienced sudden cardiac arrest (SCA). The patient had a history of marijuana consumption and was also diagnosed with a congenital ventricular septal defect (VSD) with no prior intervention or treatment. VSD is a common acyanotic congenital heart disease, which poses a constant risk of premature ventricular contractions (PVCs). During the evaluation, the patient's electrocardiogram PVCs and a prolonged QT interval were revealed. This study highlights the risk associated with the administration or consumption of drugs that can prolong the QT interval in patients with VSD. It also indicates that patients with VSD and who have a history of marijuana consumption should be cautioned about the risk of arrhythmias causing SCA due to prolonged QT interval caused by the cannabinoid. This case emphasizes the requirement of cardiac health monitoring in individuals with VSD and caution while prescribing medications that can affect the QT interval leading to life-threatening arrhythmias.

## Introduction

Ventricular septal defect (VSD) is the most common congenital heart disease that affects the ventricular muscle. With an incidence of 1-2% of live newborns being affected by moderate or severe types. Patients with VSD are known to have a higher risk of developing premature ventricular contractions (PVCs), which are abnormal heartbeats that can lead to serious arrhythmias because right ventricular pressure induces cardiac remodeling and myocyte automaticity [1]. 

Furthermore, the use of marijuana or cannabinoids has been linked to prolonging the QT interval in certain susceptible populations [2]. This prolonged QT interval can lead to a dangerous Ventricular arrhythmia called Torsade's de Pointes, which can result in cardiac arrest [3]. When these conditions; VSD, PVCs, and prolonged QT interval occur together, the risk of cardiac arrest increases significantly. Early prevention, detection, and proper management of these conditions are critical in preventing detrimental events and improving patient outcomes.

## Case presentation

**Figure 1:**
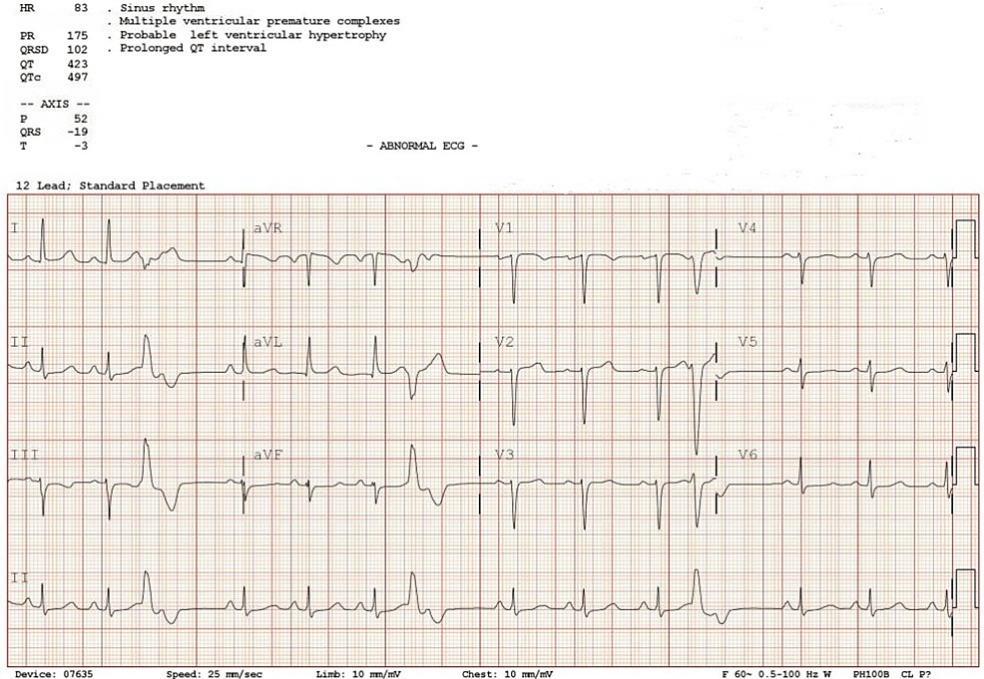
PVC (ventricular quadrigeminy) with prolonged QT interval of approximately 497ms.

A 28-year-old female was brought to the emergency department (ED) after she was found pulseless and unresponsive at home. The mother reported that the patient was playing video games, and suddenly became nonverbal and slumped. The mother began cardiopulmonary resuscitation (CPR) and return of spontaneous circulation (ROSC) was achieved after two to five minutes, after which the patient urinated and defecated on herself. The patient reported that she felt like she fell asleep and woke up with her mother screaming on top of her. In the emergency department (ED), the patient was in no acute distress but complained of chest pain, back pain, and generalized weakness. The patient had a past medical history of unrepaired ventricular septal defect (VSD), excessive marijuana consumption, cholecystectomy, and asthma. The mother reported that the reason why the VSD wasn’t repaired when the patient was born was because the VSD was noted to be small. Given that the patient was found unresponsive and pulseless at home, the ED came up with the possible differential diagnosis of ventricular arrhythmia with cardiogenic syncope, seizure, or pulmonary embolism. The patient denies any history of seizure or cigarette smoking except for daily marijuana use, unable to quantify the amount per day but indicated significant usage. Upon arrival at the ED, blood pressure was 108/78mmHg, heart rate was 86 beats per minute (BPM), and Electrocardiography (EKG) showed premature ventricular contractions (PVCs) with prolonged QT interval (Figure 1). The patient was given 1L of fluid bolus, 2g of magnesium, and 1g of calcium gluconate. Chest x-ray was significant for right lung base atelectasis vs infiltrates, normal heart size, and mediastinum. Computed Tomography (CT) of the head was unremarkable. A basic metabolic panel (BMP) and complete blood count (CBC) were performed (Table 1). 

**Table 1:** Basic Laboratories with Urine toxicology, High Sensitivity Troponin and D-dimer level.

Hematology		Chemistry	
WBC	5.41x 1000/mm^3 ^(4.5-11 NL)	Urine toxicology	Positive THC
Hemoglobin	13.0 g/dL (12-15/Adult female NL)	High sensitivity troponin	77.9 ng/mL (0-0.04 NL)
Haematocrit	37.9 % (41-50 NL)	Calcium	8.7 mg/dL (8.6- 10.3NL)
MCV	85.7 fL (80-10 NL)	Sodium	141 mEq/L (136-145 NL)
MCH	29.3 % (26- 33 NL)	Potassium	4.0 mEq/L (3.5-5.2 NL)
Platelet count	235 x 1000/mm^3 ^(150-450 NL)	Chloride	110 mmol/L (96-106 NL)
RBC	4.43 x 10^6 ^(3.8- 5.2/ Female NL)	CO_2_	22 mEq/L (23-29 NL)
D-dimer	612.97 ng/mL FEU (220-500 NL)	Blood urea nitrogen	7 mg/dL (7-20 NL)
		Creatinine	0.71 mg/dL (0.7-1.3 NL)
		Glucose	130 mmol/L (70-100 NL)
		Lactic Acid	1.2 mmol/L (0-2 NL)
		Magnesium	1.7 mg/dL (1.7-2.2 NL)
		Phosphorus	2.7 mg/dL (3.4-4.5 NL)
		TSH	1.73 mlU/L (0.5-5 NL)
		T4	0.83 µg/dL (0.9-2.3 NL)

**Figure 2:**
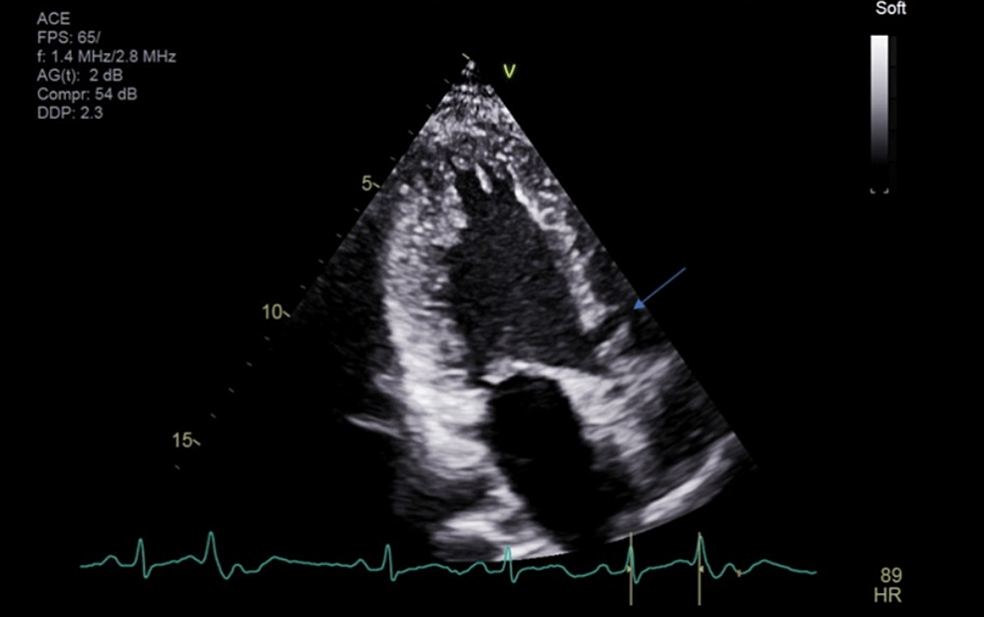
Echocardiogram – left Ventricular ejection fraction 55-60%. Subpulmonic VSD with Left to Right Shunt.

**Figure 3:**
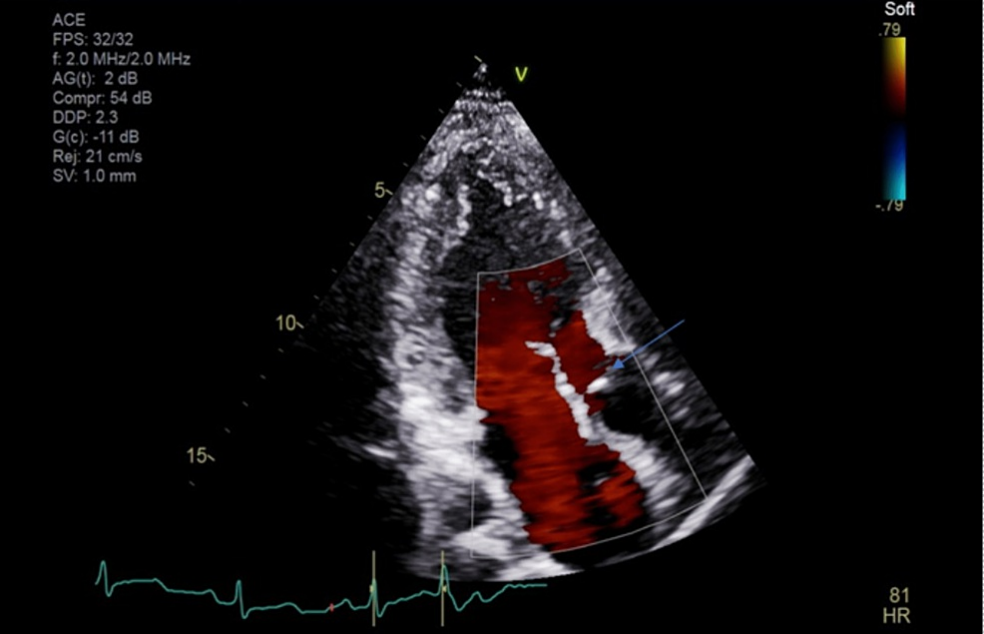
Echocardiogram with doppler – left Ventricular ejection fraction 55-60%. Subpulmonic VSD with Left to Right Shunt.

**Figure 4:**
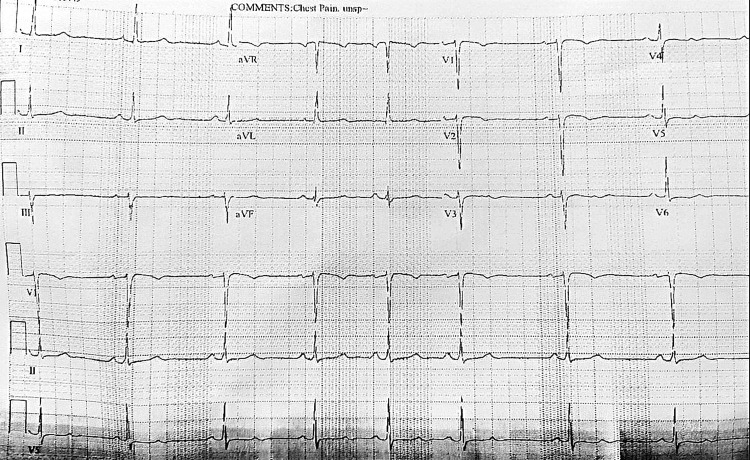
EKG – after ablation and ICD placement. No PVCs and normal sinus rhythm. EKG: electrocardiogram; ICD: implantable cardioverter-defibrillator; PVC: premature ventricular complexes.

A cardiologist was consulted post echocardiography (Figures 2, 3). There was concern of cardiac arrest caused by ventricular tachycardia induced by prolonged QT interval and multiple PVCs. Unclear if long QTc was an acquired or congenital abnormality. She was transferred to a tertiary center for further workup. In the tertiary center coronary artery CT angiogram showed a calcium score of 0, pseudoaneurysm of membranous septum measuring 12mm x 7mm without any coronary anomalies which is a new finding from the CT angiogram. Cardiac MRI showed no infiltrative process, EF 52%, Qp: Qs 1.5, and subtle mid-wall enhancement of the basal septum. Electroencephalogram (EEG) was performed and possible seizure activity was ruled out. Electrophysiology study was significant for inducible PVCs localized to the free wall right ventricular outflow tract (RVOT), ablated successfully. The team implanted an implantable cardioverter-defibrillator (ICD). A repeat electrocardiogram (EKG) was performed (Figure 4). She was discharged with nadolol 40mg daily and advised against the usage of marijuana. Long QT syndrome genetic panel sent and result pending.

## Discussion

Cardiac arrhythmias are often seen in congenital heart diseases and the incidence increases with age. A ventricular septal defect (VSD) is a congenital heart defect with an abnormal opening in the wall that separates the two ventricles of the heart. The opening allows blood to flow from the left ventricle to the right ventricle, thus increasing the right ventricular pressure. This increase in pressure can alter the electrical activity of the heart leading to the development of premature ventricular complexes (PVCs) [1]. The exact mechanism by which VSD can induce PVCs is likely due to hemodynamic and mechanical changes in the ventricular muscles and conducting system of the heart. As a result, increased blood flow and pressure in the right ventricle can cause the thickening of ventricular muscles and disruption of electrical signals creating automaticity which induces PVCs [4, 5]. In this case, Electrophysiology (EP) study revealed malignant PVCs that were easily induced during activation mapping. Activation mapping while ventricular pacing showed that the entire right ventricular outflow tract (RVOT) up to and above the pulmonic valve indicated PVCs at the anteroseptal RVOT. In unrepaired VSDs isolated PVCs, couplets, and multiform PVCs are prevalent, and non-sustained or sustained ventricular tachycardia have been observed in 6% of the cases [6].

In this report, the patient’s mother reported that the patient’s VSD was not closed due to being noted as small. A prospective study carried out from October 1976 to December 2007, explored and evaluated 187 cases of small VSD with long-term follow-up of 155 patients. This study was conducted to determine if surgical intervention is required in this patient demographic [7]. The result indicated that although a majority of the patients reported a murmur during the first month of life, spontaneous closure was seen in the first year of life in 48 cases, and within one to 5.5 years in 15 patients. With a maximum time noted as 18 years. Although persistent defects were reported until 40 years of age. The study concluded that small VSDs do not require surgical intervention (closure) as the probability of closure is approximately 34.38% in one year and 49.89% in five years [7].

The potential advantages of cannabis use have been primarily studied in the context of mental health, with less attention paid to its impact on cardiovascular health. However, as cannabinoid use becomes more prevalent in the United States, it is important to educate patients about the potential risks it may pose to their cardiovascular health given significant risk factors and excessive use. Marijuana is not just tetrahydrocannabinol (THC), it contains close to 500 compounds, and as such usage involves ingesting a lot of chemical content along with it [8]. Specific receptors for THC have been discovered in the human body. Cannabinoid receptors CB-1 and CB2 together are called endocannabinoid receptors (ECS) and are involved in cellular processes like cell proliferation and differentiation. These receptors are found in heart tissue and are also noted to regulate heart rate and blood pressure. Several studies have suggested that CB1 and CB2 receptors contribute to cardiometabolic risk and atherogenesis. CB-2 receptors have been identified in cardiomyocytes, coronary endothelial cells, and smooth muscle cells [9]. The stimulation of CB-1 and CB-2 receptor have been shown to modulate the formation of atheroma [10]. ECS has a paradoxical effect. As both receptors are stimulated, the CB-1 receptor has pro-inflammatory effects while CB-2 receptors cause anti-inflammatory and anti-atherogenic abilities. This antagonist effect is called marijuana paradox-smoking [8, 9]. This effect can decrease anginal symptoms but can trigger coronary artery events. These effects are dose-dependent. ECS is also found in the platelet membrane and high dose concentrations of cannabinoids can induce non-reversible platelet aggregation causing thrombus formation [8].

In this case report, our patient was evaluated using coronary computed tomography (CT) calcium scan to measure if there were any calcium deposits in the wall of the coronary arteries. With high calcium deposits indicate atherosclerosis, a plaque deposition in the arteries leading to heart disease. Given that marijuana can cause the formation of atheroma, oxidative stress, and platelet aggregation resulting in thrombus formation, it was imperative that our patient was evaluated with this modality. Although her score was zero, a coronary CT calcium scan can also detect coronary artery anomalies (CAA). CAA is rare, however, indicated as the leading cause of malignant arrhythmias, ischemia, and myocardial dysfunction [10]. A patient with CAA will have a high calcium score and in young patients is the second most common cause of sudden cardiac death (SCD). Our patient presented with sudden cardiac arrest (SCA), which made it important to evaluate her with a coronary CT calcium scan.

Prolonged QT can cause PVCs via a mechanism known as after-depolarizations. After-depolarizations can trigger an early depolarization of the ventricles, causing a PVC that occurs before the normal heartbeat. Thereby disrupting the normal rhythm of the heart and can potentially lead to more serious arrhythmias, including Torsade's de Pointes and ventricular fibrillation. These are life-threatening conditions that can cause SCD [11]. In susceptible patients, the use of marijuana can cause prolonged QTc leading to torsade's de pointes, ventricular tachycardia (VT), and cardiac arrest [3].

## Conclusions

Ventricular septal defect (VSD) is the most common cause of acyanotic heart disease. It is characterized by an opening in the heart wall that separates the heart's ventricles. Patients with this condition may experience premature ventricular contractions (PVCs). These occur because of changes in the automaticity of the ventricular muscle caused by the remodeling of the myocytes. Marijuana usage can lead to prolonged QT intervals in these individuals. Individuals with congenital or acquired VSD, PVCs, and prolonged QT interval are at increased risk of life-threatening arrhythmia. This study affirms that it is essential for physicians to be aware of this complication in the susceptible demographic and advise patients with VSD to avoid using any substances or medications that could potentially lengthen the QT interval. As this could lead to severe heart rhythm disturbances that could be life-threatening.
